# Comparing the effect of transcranial random noise stimulation and transcranial direct current stimulation over the motor cortex on motor performance in men vs. women: a randomized controlled crossover study

**DOI:** 10.3389/fnhum.2025.1577899

**Published:** 2025-08-08

**Authors:** Elchanan Frankel, Jason Friedman, Silvi Frenkel-Toledo

**Affiliations:** ^1^Department of Physical Therapy, School of Health Sciences, Ariel University, Ariel, Israel; ^2^Department of Physical Therapy, Stanley Steyer School of Health Professions, Gray Faculty of Medical and Health Sciences, Tel Aviv University, Tel Aviv, Israel; ^3^Sagol School of Neuroscience, Tel Aviv University, Tel Aviv, Israel; ^4^Department of Neurological Rehabilitation, Loewenstein Medical Rehabilitation Center, Raanana, Israel

**Keywords:** tRNS, tDCS, sex, motor performance, upper limb

## Abstract

Non-invasive Brain Stimulation may modulate motor function. One commonly investigated method is transcranial direct current stimulation (tDCS). In the last few years, a new stimulation technique has been developed and studied, namely transcranial random noise stimulation (tRNS). Both stimulation techniques have displayed a certain degree of inconsistency regarding their impact on motor performance. One explanation for this may be related to differences in the sex of the participants. Thirty healthy individuals (15 female) participated in a single-blind counterbalanced crossover trial. All participants received three stimulation conditions: high frequency-tRNS, tDCS, and sham stimulation. Stimulation was applied for 10 min at 1.0 mA, with a frequency range of 101–640 Hz for the tRNS. In all stimulation conditions, the anode (for tDCS) was placed over C4 and the cathode over the contralateral orbit. The participants performed a sequential reaching motor task on a digital tablet before, during, and immediately after the stimulation. Movement time, reaction time, and peak velocity did not differ between stimulation conditions. However, within-condition analyses showed improvements in movement time and peak velocity following tRNS only, while reaction time improved in all stimulation conditions. No significant effect of sex was observed. While no clear advantage for a specific stimulation condition was statistically confirmed, these within-condition effects suggest that tRNS may modestly enhance motor performance and warrant further investigation. Additionally, in this experimental setup, sex did not influence the effects of tRNS and tDCS on motor performance.

## 1 Introduction

The development of non-invasive methods for modulating neuroplasticity to improve functions is a major goal of clinical neuroscience (Peters et al., 2016). One of the main studied questions in this context is whether non-invasive brain stimulation (NIBS) techniques can be used to improve motor performance in healthy individuals (Patel et al., 2019; Bastani and Jaberzadeh, 2012) as well as people with neurological conditions (Kang et al., 2016; Van Hoornweder et al., 2021). The general assumption is that NIBS increases excitability in the motor cortex (Pascual-Leone et al., 1994; Nitsche and Paulus, 2000), which in turn could facilitate motor ability (Reis and Fritsch, 2011).

One non-invasive and painless stimulation method is transcranial direct current stimulation (tDCS; Dubljević et al., 2014) which delivers weak direct currents (usually 0.5–2 mA) through surface electrodes placed on the head. It alters cortical excitability by inducing subthreshold polarization of neuronal membranes (Nitsche and Paulus, 2000). The motor excitability levels [measured using motor evoked potentials (MEP)] may be increased or decreased among healthy participants by applying anodal (atDCS) or cathodal (ctDCS) tDCS, respectively. The effects of tDCS are complex such that anodal and cathodal stimulation can either increase or decrease cortical excitability, depending on stimulation protocols, including stimulation parameters, target regions, and electrodes montage (Nitsche and Paulus, 2000; Stagg et al., 2018; Batsikadze et al., 2013; Moliadze et al., 2015; Strube et al., 2016; Ehrhardt et al., 2021, Lerner et al., 2021; Masina et al., 2021; Vergallito et al., 2022). tDCS has been shown in some studies, but not all, to be an effective means to improve upper limb (UL) motor performance in healthy individuals (Sánchez-Kuhn et al., 2017; Patel et al., 2019) and people with stroke (Sánchez-Kuhn et al., 2017; Van Hoornweder et al., 2021). A recent meta-analysis regarding tDCS effects in healthy participants showed an improvement in UL motor performance, as demonstrated in variables such as reaction time and task completion time (Patel et al., 2019). Recent meta-analyses among people with stroke showed conflicting results with respect to the effects on both upper and lower limb motor function (Butler et al., 2013; Kang et al., 2016; Tedesco Triccas et al., 2016; O’Brien et al., 2018; Elsner et al., 2020).

Another related non-invasive method is transcranial random noise stimulation (tRNS) (Terney et al., 2008). This technique uses identical electrodes to tDCS but rather than applying a constant current, a random electrical oscillation spectrum is applied over the motor cortex. The current intensity of the electrical stimulation is generally weak, between 1.0 and 2.0 mA (Terney et al., 2008; Hayward et al., 2017; Abe et al., 2019; Arnao et al., 2019; Moret et al., 2019; Kortuem et al., 2019; Monastero et al., 2020; Splittgerber et al., 2020; Hoshi et al., 2021), and the frequency is randomly applied, with a normal bell-curve distribution within the range of 0.1–640 Hz. tRNS can be divided into two “sub-protocols”: low (0.1–100 Hz) and high (101–640 Hz) frequency (Nitsche and Paulus, 2000; Terney et al., 2008). Among healthy participants, high-frequency tRNS (hf-tRNS) has been shown to significantly increase MEP compared to sham, while low-frequency tRNS did not affect it (Terney et al., 2008). A wide range of hf-tRNS seems to be required, as frequencies of 100–400 Hz and 400–700 Hz did not modulate differently the MEP, while a full range of 100–700 Hz did (Moret et al., 2019). A proposed mechanism is that tRNS shortens the hyperpolarization phase by inducing a repetitive opening of the Na^+^ channels (Terney et al., 2008; Chaieb et al., 2015). Other proposed mechanisms include reduction of GABA levels (Sánchez-León et al., 2021) and stochastic resonance (Terney et al., 2008; Pavan et al., 2019). The latter relates to a phenomenon in which the addition of random interference (noise) in a non-linear system can enhance the detection of weak signals or enhance the information content of a signal (Moss et al., 2004; McDonnell and Abbott, 2009). An optimal dose of noise can lead to peak enhancement of the information content, but further noise will lead to degradation of the content or reduce its detectability (Moss et al., 2004).

Compared to the multitude of studies on tDCS and its effect on motor performance and corticospinal excitability in healthy and people with neurological conditions (Broeder et al., 2015, Kang et al., 2016, Sánchez-Kuhn et al., 2017; Patel et al., 2019), there is relatively limited research on the effects of tRNS on motor performance in both healthy individuals (Terney et al., 2008; Saiote et al., 2013; Prichard et al., 2014; Abe et al., 2019; Jooss et al., 2019; Brevet-Aeby et al., 2019; Hoshi et al., 2021) and people with neurological conditions (Hayward et al., 2017; Arnao et al., 2019; Monastero et al., 2020), with a greater focus on its impact on MEP (Chaieb et al., 2011; Moliadze et al., 2012; Laczó et al., 2014; Chaieb et al., 2015; Ho et al., 2015; Inukai et al., 2016; Kortuem et al., 2019; Moret et al., 2019; Haeckert et al., 2020; Splittgerber et al., 2020; Zhang et al., 2021). Only several studies investigated the effect of tRNS over primary motor cortex (M1) on motor performance in healthy participants (Terney et al., 2008; Saiote et al., 2013; Prichard et al., 2014; Abe et al., 2019; Jooss et al., 2019; Hoshi et al., 2021). Some tRNS studies showed significant motor improvement (but not all, Hoshi et al., 2021), as indicated by reduced reaction times (Terney et al., 2008; Jooss et al., 2019), decreased error rates (Abe et al., 2019), and increased accuracy in UL motor performance (Jooss et al., 2019). Among people with stroke, findings regarding the effect on UL motor ability are inconsistent (Hayward et al., 2017; Arnao et al., 2019).

A comparison between the neurophysiological and behavioral effects of tDCS and tRNS has been done in several domains, such as pain perception (Yao et al., 2021), numerical cognition (Bieck et al., 2018), visual perceptive learning (Herpich et al., 2019), and visuomotor learning (Saiote et al., 2013). To the best of our knowledge, only two studies compared the effects of tDCS and tRNS on UL motor performance (Saiote et al., 2013; Prichard et al., 2014), and some others compared their effects on MEP (Moliadze et al., 2014; Ho et al., 2015; Inukai et al., 2016; Haeckert et al., 2020) and phosphene thresholds as a measure of visual cortex excitability (Herpich et al., 2018). From a behavioral perspective, both hf-tRNS and ctDCS over M1 showed a trend toward improvements in error rates, while lf-tRNS exhibited a trend toward hindering error rates in a visuomotor task. No effects were observed for atDCS or sham treatment (Saiote et al., 2013). Prichard et al. (2014) found that unilateral M1 tRNS and unilateral and bilateral M1 tDCS enhanced motor skill learning compared to sham stimulation. While unilateral tDCS produced substantial skill gains immediately following the stimulation, tRNS had a more gradual impact. From a neurophysiological aspect, when comparing the effects of 1 mA tDCS and full-spectrum tRNS (0.1–640 Hz) with sham stimulation at 0, 5, 10, and 20 min post-stimulation on MEP, only tRNS presented significant increases in MEP compared to sham (Inukai et al., 2016). Also, Haeckert et al. (2020) found that in healthy individuals, hf-tRNS lasting 7 min and 13 min resulted in increased MEP amplitudes, but in contrast tDCS did not present any significant changes. Interestingly, Ho et al. (2015) showed significant increases in MEP from baseline after 1 and 2 mA tDCS and after 2 mA hf-tRNS with a DC offset of 1, but not after 2 mA hf-tRNS with no offset.

As mentioned, the results of tDCS and tRNS studies aiming to improve UL motor performance in healthy and/or post-stroke participants by targeting M1 have been inconsistent (Bastani and Jaberzadeh, 2012; Abe et al., 2019; Patel et al., 2019; Elsner et al., 2020; Hoshi et al., 2021). One possible reason may relate to the fact that sex was not taken into consideration in most NIBS studies as a factor that can influence the response to the stimulation (Kuo et al., 2006). Sex was found to mediate the effects of NIBS on cortical induced electric field current (Russell et al., 2014; Thomas et al., 2019), and on different behaviors (Kuo et al., 2006; Gorbet and Staines, 2011; Adenzato et al., 2019; Fehring et al., 2021) such as visually guided reaching movements (Gorbet and Staines, 2011) and social cognition skills (Adenzato et al., 2019). The responsiveness to tDCS may be related to variances in hormonal levels (Krause and Cohen Kadosh, 2014; Rudroff et al., 2020), neurotransmitter balances and anatomical bone density (Russell et al., 2014; Rudroff et al., 2020) between sexes. Hormonal levels fluctuate significantly more in women than in men (Krause and Cohen Kadosh, 2014; Rudroff et al., 2020). Progesterone appears to drive the increase of cortical inhibition, likely through gamma-aminobutyric acid (GABA), and estradiol enhances brain excitability (Smith et al., 2002; Inghilleri et al., 2004; Rudroff et al., 2020), likely through glutamatergic mechanisms (Hanlon and McCalley, 2022). Modulations in GABA concentrations induced by active tDCS have been linked to individual differences in motor learning capacity (Kim et al., 2014). With regard to bone density, different studies have shown conflicting results as to whether men or women receive more electrical current from tDCS (Russell et al., 2014, 2017; Thomas et al., 2019). Indeed, studies have indicated sex-related anatomical differences in head and brain structures (Amunts et al., 1996; Ruigrok et al., 2014; Gennatas et al., 2017). For instance, modeling study showed that young males (but not middle-age and old-age groups) had a higher current density than females, only for the parietal and not frontal montage (Bhattacharjee et al., 2022), possibly due to more porous bone (a thicker spongy layer) in males than females, especially in the parietal rather than the frontal bone (Rampersad et al., 2011). Sex related differences in gray and white matter among healthy individuals may contribute to differential responses to brain stimulation. Women exhibit a higher gyrification index in frontal and parietal cortices, reflecting increased cortical folding and greater gyral surface area (Luders et al., 2004). After controlling for total intracranial volume, women exhibited greater frontal and parietal cortex volume than men (Hanlon and McCalley, 2022). Unlike tDCS, the effect of sex on response to tRNS has been scarcely explored (Manippa et al., 2017) and has not been investigated in the context of the motor domain.

This study is the first attempt to compare the effects of tDCS vs. tRNS on UL motor ability while taking into consideration the effect of sex in healthy subjects. Previous findings generally have shown equal (Saiote et al., 2013; Ho et al., 2015; Yao et al., 2021) or better (Inukai et al., 2016; Herpich et al., 2019; Haeckert et al., 2020) results following tRNS compared to tDCS in MEP across other domains (e.g., pain perception, visual perception). Therefore, we hypothesized that motor performance would improve after both hf-tRNS and tDCS compared to sham stimulation, with tRNS showing greater efficacy than tDCS. Furthermore, we expected the participants’ sex would mediate the effects of tDCS and tRNS. The exact effect is unclear, as different studies present different effects (Manippa et al., 2017; Yang and Banissy, 2017). Such a comparison, while relating to participants’ sex, may clarify the effects of tDCS vs. tRNS on UL motor performance.

## 2 Materials and methods

### 2.1 Study design

This was a single-blind counterbalanced crossover study in which all participants received three stimulation conditions with a 1-week wash-out break (Abe et al., 2019; Jooss et al., 2019; Kortuem et al., 2019; Monastero et al., 2020; Hoshi et al., 2021) between each condition. The conditions were: (1) hf-tRNS, (2) tDCS, and (3) sham stimulation. The order of the stimulation techniques was counterbalanced within each sex. To ensure the blinding of participants, the stimulator monitor was hidden from the participants, and the sham stimulation increased and decreased in a ramp-like fashion at the beginning and end of the stimulation period. Data was collected in a brain and motor behavior laboratory based at Ariel University.

### 2.2 Participants

**FIGURE 1:**
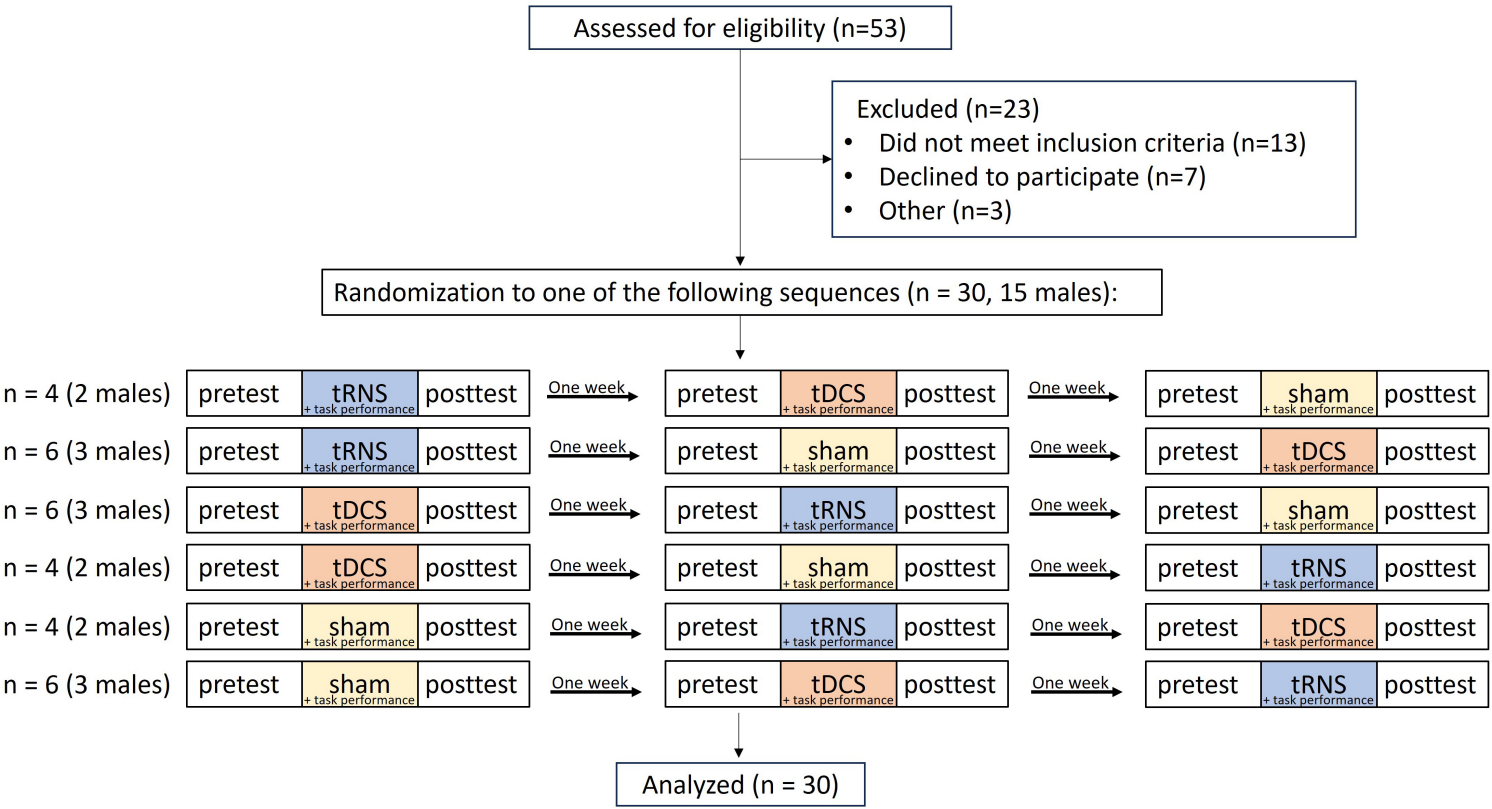
Trial flowchart. tRNS, transcranial random noise stimulation; tDCS, transcranial direct current stimulation.

The sample size for this study was determined based on a power analysis calculation that was conducted using G*Power version 3.1.9.7. Power analysis yielded a total sample size of 28 individuals for the detection of a significant interaction with an assumed effect size of 0.25 and a power of 95%. To account for potential data loss, we aimed for a sample size of 30 individuals. The flowchart illustrating the study procedure can be found in Figure 1. Fifty-three participants underwent the pre-enrollment screening evaluation. Of those, 13 did not meet the inclusion criteria, 7 chose not to participate, and 3 were excluded due to technical difficulties. Thirty participants (15 females, 15 males) participated in the study, with an average age of 24.53 ± 2.37 years, recruited via convenience sampling. Participants were included if they were between the ages 20–35, were right-hand dominant, according to the Edinburgh questionnaire (Oldfield, 1971), and self-reported as healthy. Participants were excluded if they were diagnosed with a neurological or psychiatric disorder, had a history of drug abuse, had metal implants in their head, or used psychiatric medications. Participants signed an informed consent form prior to participating in the study. All procedures were approved by the Ariel University Institutional Ethical Board (approval number: AU-HEA-SFT-20220808) and were performed in accordance with relevant guidelines and regulations. Participants were paid $40 for their participation.

### 2.3 Stimulation

**FIGURE 2:**
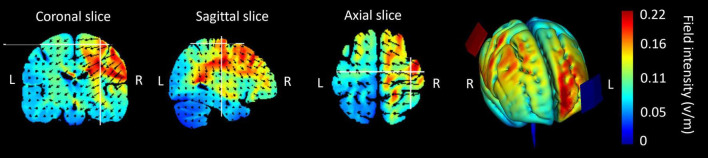
Current flow modeling during 1 mA transcranial direct current stimulation (tDCS) using the Explore software (Soterix Medical, New York, NY). Current-flow models of the right primary motor cortex (M1) are shown on 2D and 3D reconstructions of the cortical surface. Skin, skull, and cerebrospinal fluid (CSF) masks are suppressed to reveal the underlying gray matter mask. A head model derived from the MNI 152 dataset was used. The spatial profile of the current flow map is identical to that of tDCS; however, the field intensity is halved for the 1.0 mA peak-to-peak hf-tRNS.

The stimulation was administered by a battery driven electrical stimulator (DC-Stimulator Plus, neuroConn) through conductive rubber electrodes placed in two saline-soaked sponges (5 × 7 cm). The stimulation conditions were 1.0 mA (current density: 0.286 A/m^2^) tDCS (Inukai et al., 2016), 1.0 mA peak-to-peak hf-tRNS (current density: 0.143 A/m^2^) with a range of 101–640 Hz (Terney et al., 2008; Inukai et al., 2016; Abe et al., 2019; Hoshi et al., 2021) with no DC offset, and sham stimulation, as these parameters have been shown to have positive effects on MEP. All stimulation conditions were applied for 10 min. In tDCS and hf-tRNS conditions, the current increased and decreased in a ramp-like fashion over the course of the first and last 30 s, respectively. In the sham condition, a tDCS current was ramped up to 1 mA over the first 30 s and ramped back down over the following 30 s. In the last minute of the simulation, an identical ramp up and ramp down occurred (for a similar approach, see Charvet et al., 2018; Lerner et al., 2021). In all stimulation conditions, the target electrode was placed over C4 (anode in the case of tDCS) using the electroencephalogram (EEG) 10–20 referencing system with the reference over the contralateral orbit (cathode in the case of tDCS). This electrode configuration has been employed in previous studies involving tDCS (e.g., Nitsche and Paulus, 2000; Bastani and Jaberzadeh, 2012; Ehrhardt et al., 2021) and tRNS (e.g., Terney et al., 2008; Jooss et al., 2019; Hoshi et al., 2021). HD-Explore brain modeling software (Soterix Medical, New York, NY) was used to determine the tDCS montage for maximal focal stimulation of the right M1 (Figure 2). Participants were asked to report any adverse effects and to rank their discomfort from 1 to 10 one min after the stimulation began.

### 2.4 Motor task

**FIGURE 3:**
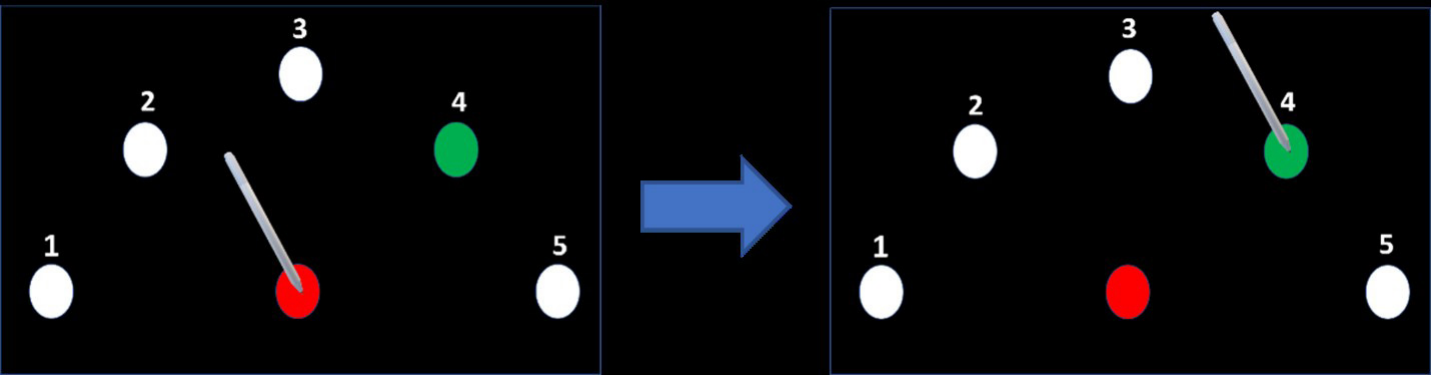
Motor task. After placing the stylus on the center target (starting point) and remaining there for 200 ms, the center target’s color turned red and the color of one of the targets in the semicircle turned green, according to a predefined sequence. The participant dragged the pen on the target to the green target and held it there until its color returned to white. Note that for clarity the size of the targets shown in the figures are much larger than the targets used in the experiment.

In all participants, the non-dominant left arm was tested. After placing the electrodes on the head, the participants performed a sequential point-to-point movement task on a graphics tablet, a version of a similar, previously used task (Ghilardi et al., 2000; Ghilardi et al., 2009; Moisello et al., 2009; Lerner et al., 2021; Swissa et al., 2022). The stimuli consisted of a starting point and five targets equally spaced around it in a semicircle, all equidistant from the starting point (17 cm) and all with a diameter of 0.5 cm (Figure 3). Each movement began at the starting point. After holding the stylus at the starting point for 200 ms, the starting point changed its color from white to red, and one of the targets changed color from white to green, after which the participants needed to drag the stylus to the green target. They needed to remain there for 500 ms (until the target returned to its initial color), then lift the stylus and return it to the starting position to start the next movement. In each session, the participants were instructed that the targets would follow one of the three sequences 4-1-3-2-5, 5-2-3-1-4, 1-4-2-3-5, and to perform the task as fast and accurately as possible. The order of the sequences between conditions was counterbalanced among participants.

During each session, participants underwent the following procedure for each of the three conditions (tRNS, tDCS, sham). Initially, the participants performed the motor task until they successfully completed 3 sequences without errors to familiarize themselves with the task. Then, they performed the pretest, which consisted of one block of 6 sequences (i.e., 30 movements). Upon completion, the stimulation was activated. One minute after starting the appropriate stimulation, the participants were asked about adverse effects. They then performed 3 blocks of 6 sequences, with a 30-s break between each block. After finishing the tDCS/hf-tRNS/sham stimulation, the participants performed an immediate post-test, which was identical to the pretest.

Three outcome measures were used: movement time (MT) (s), reaction time (RT) (s), and peak velocity (PV) (cm/s). Movement time was defined as the time from movement onset (first time the tangential velocity was greater than 5% of the peak tangential velocity) until the end of movement (last time the tangential velocity was greater than 5% of the peak tangential velocity). RT was defined as the time from the moment the target in the semicircle turned green until movement onset. PV was defined as the maximum tangential velocity achieved during the movement. Improved motor performance was indicated by a shorter MT, a shorter RT, and a higher PV.

### 2.5 Statistical analysis

For the kinematic measures, the assumption of a normal distribution was determined using the Kolmogorov–Smirnov test. Since RT values were not normally distributed, they were log-transformed before this analysis (the original values are presented for clarity). A two-way repeated measures-ANOVA (RM-ANOVA) was used with time (pretest, posttest) and stimulation condition (tRNS, tDCS, sham) as within-subject factors. Sex (male, female) was added as a between-subject factor. The Bonferroni correction was used when there were multiple comparisons. The Greenhouse–Geisser Epsilon (G-GE) was used to correct the degrees of freedom when Mauchly’s test of sphericity was significant. Differences between stimulation conditions were also investigated by comparing delta values between timepoints (calculated by subtracting pretest scores from posttest scores for each participant) using a RM-ANOVA with stimulation condition (tRNS, tDCS, sham) as within-subject factors and sex (male, female) as a between-subject factor. The Bonferroni correction was used when there were multiple comparisons. The differences between conditions with respect to the frequency of adverse effects were tested using a Cochran’s Q test with *post hoc* Dunn’s test (when necessary) and with Bonferroni correction for multiple comparisons. The differences between conditions with respect to the discomfort from adverse effects were calculated using Friedman’s test with *post hoc* Wilcoxon signed-rank tests (if necessary) with Bonferroni correction for multiple comparisons. All tests were performed using SPSS (version 29.0) with initial significance levels of *p* < 0.05.

## 3 Results

Mean values of MT (s), RT (s) and PV (cm/s) are displayed in Table 1. Movement time, RT and PV did not differ at pretest between stimulation conditions (*p* > 0.074, for all).

**TABLE 1:** Means, standard deviations and confidence intervals of movement time, reaction time and peak velocity for stimulation conditions at pretest and posttest.

Variable	hf-tRNS 1.0 mA (*n* = 30)		tDCS 1.0 mA (*n* = 30)		Sham (*n* = 30)	
	Pretest	Posttest	Pretest	Posttest	Pretest	Posttest
Movement time (s): mean ± SD [CI]	1.05 ± 0.33 [0.93–1.17]	0.96 ± 0.29 [0.86–1.07]	0.96 ± 0.26 [0.87–1.06]	0.93 ± 0.24 [0.84–1.01]	0.98 ± 0.32 [0.87–1.1]	0.95 ± 0.28 [0.85–1.06]
Reaction time (s): mean ± SD [CI]	0.86 ± 0.21 [0.78–0.94]	0.78 ± 0.12 [0.73–0.81]	0.82 ± 0.13 [0.77–0.87]	0.76 ± 0.11 [0.72–0.8]	0.86 ± 0.2 [0.79–0.94]	0.8 ± 0.18 [0.73–0.87]
Peak velocity (cm/s) mean ± SD [CI]	36.03 ± 10.43 [32.33–39.73]	38.51 ± 10.62 [34.77–42.24]	38.03 ± 9.36 [34.7–41.35]	39.15 ± 9.14 [35.93–42.36]	38.53 ± 10.77 [34.68–42.39]	38.81 ± 10.29 [34.96–42.66]

Hf-tRNS, high-frequency transcranial random noise stimulation; tDCS transcranial direct current stimulation; CI, 95% confidence interval.

### 3.1 Effects on movement time

**FIGURE 4:**
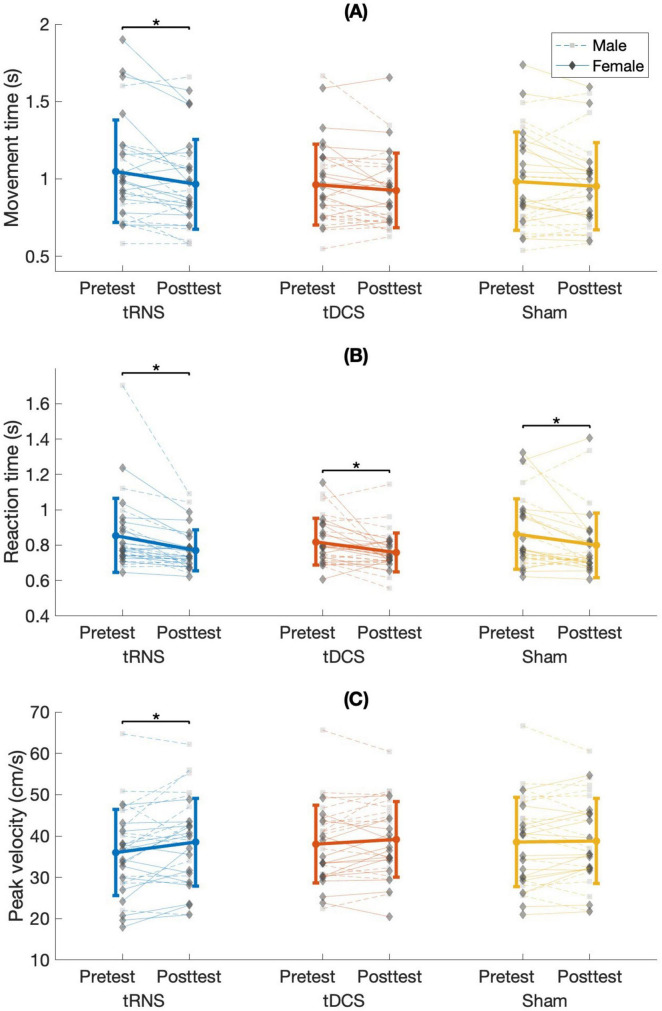
**(A)** Mean movement time, **(B)** reaction time, and **(C)** peak velocity for stimulation conditions in males and females at pretest and posttest. s, seconds; cm/s, centimeters per second; tRNS, transcranial random noise stimulation; tDCS, transcranial direct current stimulation. Error bars show the standard deviation. It is important to note that the asterisks (*) refer to comparisons between time points within each stimulation condition, which remained significant after Bonferroni correction, despite the absence of a significant Stimulation Condition × Time interaction (for justification, see Wei et al., 2012).

A main effect of time [F(1,28) = 13.281; *p* = 0.001; partial η^2^ = 0.322; observed power = 0.940] showed that across the different stimulation conditions, MT decreased significantly from pretest to posttest (1.00 ± 0.3 s, 0.95 ± 0.27 s, respectively). No significant interactions were observed [stimulation condition × sex: F(2,56) = 0.735; *p* = 0.484; time × sex: F(1,28) = 2.494; *p* = 0.126; stimulation condition × time: F(2,56) = 1.492; *p* = 0.234; stimulation condition × time × sex: F(2,56) = 1.251; *p* = 0.294]. The delta from pretest to posttest in MT did not differ significantly between stimulation conditions [F(2,56) = 1.492; *p* = 0.234; partial η^2^ = 0.051; observed power = 0.305]. To gain a more comprehensive understanding of time-related effects within each stimulation condition, we additionally examined whether MT significantly differed between time points within each condition. Therefore, despite the non-significant interaction, we further investigated the time effects within each stimulation condition. This approach aligns with the perspective of Wei et al. (2012), who highlight the value of examining condition means even in the absence of interaction effects. A similar method was applied by Jobgen et al. (2009). A significant main effect of time was found only for tRNS [F(1,28) = 9.759; *p* = 0.004 (≤ pBonferroni = 0.017); partial η^2^ = 0.258; observed power = 0.854]. MT decreased significantly from pretest to posttest (1.05 ± 0.33 s and 0.96 ± 0.29 s, respectively) (Figure 4A).

### 3.2 Effects on reaction time

A main effect of time [F(1,28) = 32.161; *p* < 0.001; partial η^2^ = 0.535; observed power = 1] showed that across the different stimulation conditions RT decreased significantly from pretest to posttest (0.85 ± 0.18 s, 0.78 ± 0.14 s, respectively). No significant interactions were observed [stimulation condition × sex: F(2,56) = 0.054; *p* = 0.947; time × sex: F(1,28) = 0.008; *p* = 0.929; stimulation condition × time: F(2,56) = 0.223; *p* = 0.801; stimulation condition × time × sex: F(2,56) = 0.412; *p* = 0.665]. The delta from pretest to posttest in RT did not differ significantly between stimulation conditions [F(2,56) = 0.377; *p* = 0.688; partial η^2^ = 0.013; observed power = 0.108]. To gain a more comprehensive understanding of time-related effects within each stimulation condition, we additionally examined whether RT significantly differed between time points within each condition. Therefore, despite the non-significant interaction, we further investigated the time effects within each stimulation condition (Wei et al., 2012; Jobgen et al., 2009). A. Significant effects of time were observed among all stimulation conditions, all presenting decreased RT from pretest to posttest: tRNS [F(2,28) = 22.147; *p* < 0.001; (≤ pBonferroni = 0.017); partial η^2^ = 0.442; observed power = 0.995. Pretest: 0.86 ± 0.21 s, posttest: 0.78 ± 0.12 s], tDCS [F(1,28) = 8.921; *p* = 0.006; (≤ pBonferroni = 0.017); partial η^2^ = 0.242; observed power = 0.822. Pretest: 0.82 ± 0.13 s, posttest: 0.76 ± 0.11 s], and sham [F(1,28) = 10.002; *p* = 0.004; (≤ pBonferroni = 0.017); partial η^2^ = 0.263; observed power = 0.863. Pretest: 0.86 ± 0.2 s, posttest: 0.8 ± 0.18 s] (Figure 4B).

### 3.3 Effects on peak velocity

A main effect of time [F(1,28) = 5.778; *p* = 0.023; partial η^2^ 0.171; observed power = 0.641] showed that across the different stimulation conditions PV increased significantly from pretest to posttest (37.53 ± 10.15 cm/s, 38.82 ± 9.93 cm/s, respectively). No significant interactions were observed [stimulation condition × sex: F(2,56) = 0.785; *p* = 0.461; time × sex: F(1,28) = 0.660; *p* = 0.423; stimulation condition × time: F(2,56) = 2.604; *p* = 0.083; stimulation condition × time × sex: F(2,56) = 2.697; *p* = 0.076]. The delta from pretest to posttest in PV did not differ significantly between stimulation conditions [F(2,56) = 2.604; *p* = 0.083; partial η^2^ = 0.085; observed power = 0.498]. To gain a more comprehensive understanding of time-related effects within each stimulation condition, we additionally examined whether PV significantly differed between time points within each condition. Therefore, despite the non-significant interaction, we further investigated the time effects within each stimulation condition (Wei et al., 2012; Jobgen et al., 2009). A significant effect of time was found for only tRNS [F(1,28) = 6.433; *p* = 0.017; (≤ pBonferroni = 0.017); partial η^2^ = 0.187; observed power = 0.687]. PV increased significantly from pretest to posttest (36.03 ± 10.43 cm/s, 38.51 ± 10.62 cm/s, respectively) (Figure 4C).

### 3.4 Adverse effects

The stimulation was well tolerated by the participants, and no sessions were aborted due to adverse effects. The frequency and level of discomfort caused by adverse effects are displayed in Table 2.

**TABLE 2:** Frequency and discomfort of adverse effects. Median values and interquartile ranges of discomfort are presented.

Adverse effect	tRNS (*n* = 30)		tDCS (*n* = 30)		Sham (*n* = 30)	
	Frequency	Discomfort	Frequency	Discomfort	Frequency	Discomfort
Tingling	2 (7%)	0 (0–3)	19 (63%)	2.5 (0–9)	23 (77%)	4 (0–7)
Itching	1 (3%)	0 (0–1)	8 (27%)	0 (0–8)	6 (20%)	0 (0–10)
Burning	0 (0%)	0 (0–0)	2 (7%)	0 (0–4)	2 (7%)	0 (0–7)
Other	2 (7%)	0 (0–3)	1 (3%)	0 (0–3)	1 (3%)	0 (0–2)

tRNS, transcranial random noise stimulation; tDCS, transcranial direct current stimulation. Median values and ranges are presented.

#### 3.4.1 Frequency

Frequency of “tingling” and “itching” significantly differed between conditions (χ^2^(2) = 32.435, *p* < 0.001, χ^2^(2) = 6.5, *p* = 0.039, respectively). “Tingling” was significantly less frequent during tRNS compared to tDCS (*p* < 0.001) and sham (*p* < 0.001). “Itching” was significantly less frequent during tRNS compared to tDCS (*p* = 0.040). No other significant effects were observed.

#### 3.4.2 Discomfort

Discomfort levels of “tingling” and “itching” significantly differed between conditions (χ^2^(2) = 30.414, *p* < 0.001, χ^2^(2) = 6.513, *p* = 0.039, respectively). Discomfort level of “tingling” was significantly lower during tRNS compared to tDCS (*Z* = −3.831; *p* < 0.001) and sham (*Z* = −4.218; *p* < 0.001). Discomfort level of “itching” was significantly lower during tRNS compared to tDCS (*Z* = −2.552; *p* = 0.011). No other significant effects were observed.

## 4 Discussion

To the best of our knowledge, this is the first study to compare the effects of tRNS vs. tDCS on motor performance in healthy participants, while taking into consideration the effects of sex. We found no significant Stimulation × Time or Stimulation × Time × Sex interactions. However, significant improvements from pretest to posttest were found in MT and PV only in the tRNS condition (RT improved in all stimulation conditions). These results do not offer statistically significant evidence to support the superiority of one stimulation condition over another. However, they provide support for a potential modest benefit of tRNS on UL motor performance. These trends warrant further investigation in the future.

Similar to our findings, Saiote et al. (2013) reported no significant Simulation × Time interaction, but they observed a tendency for 1 mA of hf-tRNS to enhance motor learning (reduced tracking error) after 10 min. Similarly, our study applied 1 mA tRNS for 10 min, using a frequency range of 101–640 Hz. In contrast, three previous studies found significant improvements in motor function following hf-tRNS to M1 compared to sham stimulation (Prichard et al., 2014; Terney et al., 2008; Abe et al., 2019). These differences may be explained by variations in stimulation parameters. For example, one of the above-mentioned studies applied stimulation for 20 min (Prichard et al., 2014) whereas the duration in our study was 10 min. Indeed, stimulation duration has been shown to modulate the effects of both tRNS (Haeckert et al., 2020) and tDCS (Monte-Silva et al., 2010; Haeckert et al., 2020). However, even among studies that employed the same stimulation parameters as ours (1 mA for 10 min), Terney et al. (2008) and Abe et al. (2019) reported enhanced motor performance following tRNS compared to sham stimulation. This variability highlights the complexity of comparing tRNS effects across studies, as outcomes are influenced by multiple interacting factors, including stimulation intensity and duration, electrode montage, and the resulting current flow patterns in the brain (Esmaeilpour et al., 2018; Peterchev et al., 2012). Additionally, individual anatomical variability can affect current density, potentially leading to variable intensity–response relationships across participants (Laakso et al., 2015). Task complexity may further shape the observed behavioral effects of stimulation, adding another layer of variability to inter-studies comparisons.

Our finding that motor performance did not improve after tDCS compared to sham stimulation contradicts our hypothesis. In addition, the variables MT and PV did not improve from pretest and posttest following tDCS. These results contradict those of a previous study that used a similar task in which MT improved at posttest as compared to pretest (Lerner et al., 2021). This discrepancy may be due to differences in studies’ protocols. The earlier study (Lerner et al., 2021) found this effect only after HD-tDCS at 1.5 mA, not at 2 mA, whereas our study applied conventional tDCS at 1.0 mA. Boggio et al. (2006), who utilized 1.0 mA conventional tDCS similarly to our study, found significant motor improvements compared to sham. However, their study differed in key aspects: they employed a different motor task (Jebsen Taylor Hand Function Test), applied stimulation for 20 min, and included a small sample size of eight participants, all of whom were female.

Unlike MT and PV which significantly improved from pretest to posttest in the tRNS condition only, RT significantly improved from pretest to posttest in all stimulation conditions. Reaction time reflects motor preparation, while MT and PV reflect aspects of movement execution. A recent meta-analysis (Kang et al., 2016) showed modest improvements in RT with smaller effect sizes than for execution time following tDCS in healthy participants (Sánchez-León et al., 2021). The primary motor cortex is primarily associated with response execution (Ghilardi et al., 2009; Moisello et al., 2009; Charvet et al., 2018), while the premotor cortex is more involved in response selection and preparation. In this study, the execution task involved sequential point-to-point movements using the non-dominant hand toward small 0.5 cm targets on a graphics tablet. The challenges and potential for improvement in the execution component of this task, combined with the stimulation site targeting M1 rather than premotor areas, may have contributed to the observed enhancements in MT and PV from pretest to posttest in the tRNS condition but not in the sham condition. Reaction time likely improved from pretest to posttest across all stimulation conditions as a result of training, aligning with findings from similar sequence learning tasks (Ghilardi et al., 2000, Lerner et al., 2021). Alternatively, the observed enhancement may reflect a generalized placebo effect associated with the stimulation conditions. Subjective expectations may induce a specific brain state that interacts with the effects of electrical stimulation. However, since participants’ expectations and beliefs were not assessed before or after the stimulation (Braga et al., 2021), it is difficult to rule out or quantify the potential contribution of placebo effects on motor performance.

The current study did not reveal a clear advantage for tRNS over tDCS or sham on any measure, as indicated by the non-significant Time × Stimulation interaction. However, as noted earlier, tRNS and not tDCS generally demonstrated significant differences between pretest and posttest. It should be noted that baseline performance across stimulation conditions was comparable for all outcome measures, minimizing the likelihood that initial stimulation conditions differences influenced these results. Similarly, Saiote et al. (2013), who compared the effects of 10 min tRNS (low frequency (lf) tRNS: 0.1–100 Hz, hf-tRNS: 101–640 Hz) and 1 mA tDCS over the left M1, found a non-significant Time × Stimulation interaction. Their findings showed a trend toward accelerated learning following cathodal tDCS and hf-tRNS, but not after atDCS, while lf-tRNS appeared to impair the learning process. It is possible that in the current study, it should be noted that the effect of tRNS on motor ability is unlikely to be attributed to placebo effects because the frequency and levels of discomfort of tingling and itching were significantly lower during tRNS compared to tDCS (see Table 2). These results are consistent with previous studies, which have shown that blinding was less effective for tDCS than tRNS (Sheffield et al., 2022).

The subtle behavioral differences between pretest and posttest in the tDCS and tRNS conditions likely reflect distinct neural mechanisms underlying these stimulation modalities. In Saiote et al. (2013)’s study, tDCS did not significantly modulate brain activity, whereas hf-tRNS was associated with reduced motor task-related activity bilaterally in the frontal cortex and precuneus, possibly due to hf-tRNS interacting with ongoing neuronal oscillations. Stochastic resonance mechanism may underline the modest but more pronounced behavioral improvements observed following tRNS compared to tDCS and sham stimulation in this study. According to this mechanism, the addition of random interference (i.e., noise) can enhance the detection of weak stimuli or enhance the information content of a signal (Pavan et al., 2019; Ward, 2009). The presence of an optimal amount of neural noise by tRNS could enhance the sensitivity of neurons to a weak stimulus (Miniussi et al., 2013). Additionally, hf-tRNS may be associated with repetitive opening of Na+ channels, thereby enhancing cortical excitability (Terney et al., 2008; Antal and Herrmann, 2016). Future studies incorporating neurophysiological measures such as EEG or TMS are warranted to directly assess cortical excitability, connectivity, and oscillatory dynamics, thereby clarifying the distinct neural mechanisms associated with each stimulation technique.

No significant Stimulation × Sex or Stimulation × Time × Sex were found in the current study. This finding contradicts our hypothesis that sex would modulate the effects of both tRNS and tDCS. It also contrasts with the results of Swissa et al. (2022), who employed a similar motor task. Swissa et al. (2022) investigated the impact of 15 min HD-tDCS over M1 at 1.0 mA on a sequential reaching motor task in men vs. women. They found a reduction in RT following HD-tDCS over M1 only in men. Such sex-related differences were not observed in the present study. Several explanations may account for the discrepancy between our results and those of Swissa et al. (2022). First, methodological differences could influence neural responsiveness, as Swissa et al. (2022) utilized a HD electrode montage and a longer stimulation duration. Second, differences in the timing of post-test measurements may explain the variable findings across studies. For example, Kuo et al. (2006) found no sex-related differences after 13 min of 1.0 mA atDCS. However, 90 min post-tDCS, they found that the excitatory effects of atDCS on MEPs persisted in men but not in women. In addition, sex-related differences in tDCS responsiveness between studies may have stemmed from hormonal fluctuations and cortical anatomy of participants, which were not controlled in the current study and Swissa et al. (2022)’s study. Menstrual cycle phase may have influenced the outcomes, as progesterone appears to increase cortical inhibition and estradiol to heightened excitability (Krause and Cohen Kadosh, 2014; Inghilleri et al., 2004; Smith et al., 2002). It is important to note that, consistent with our findings, several studies have reported that sex does not significantly influence the effects of tDCS on motor abilities (Fehring et al., 2021; Hsu et al., 2025). A recent preregistered study by Hsu et al. (2025) examined the dose–response relationship of tDCS on motor learning and cortical excitability and found no significant differences in motor performance based on sex. Furthermore, sex does not appear to mediate all aspects of tDCS-related motor outcomes. In a study by Fehring et al. (2021), tDCS applied over the dorsolateral prefrontal cortex affected response inhibition similarly in males and females, although differences emerged in response execution.

### 4.1 Limitations

The study has several limitations. First, the study was a single-blind crossover randomized controlled trial, in which the researcher, who applied the stimulation and ran the motor task, was aware of the stimulation condition. However, this potential bias was mitigated by the automatic recording and analysis of kinematic measures using the MATLAB program. Second, a common limitation to NIBS studies is the large variability in skull and brain anatomy across individuals (Laakso et al., 2015), which increases the likelihood of different responses to the stimulation. Previous studies have shown that 20%–60% of participants exhibited an excitability increase induced by a single atDCS session (Chew et al., 2015; Lopez-Alonso et al., 2014, 2015; Nuzum et al., 2016; Strube et al., 2015; Wiethoff et al., 2014). The intensity of the induced electric field has been shown to vary with gender and ethnicity, with skull thickness, scalp thickness, and the thickness of the epidural cerebrospinal fluid identified as key anatomical determinants of inter-individual electric field variability (Ma et al., 2024). While the crossover design mitigates the impact of inter-individual anatomical variability on comparisons between stimulation conditions, such anatomical factors may still influence overall responsiveness to stimulation. This may have contributed to increased between-subject variability and could have limited the ability to detect subtle differences between stimulation conditions. Future studies accounting for these factors may improve the accuracy and consistency of stimulation outcomes by minimizing individual differences. Third, a wider age range among participants would improve the external validity of the findings and enhance their applicability to a broader population. Fourth, we employed a widely used electrode montage in which the target electrode was placed over C4 (anode in the case of tDCS) and the reference electrode over the contralateral orbit (cathode in the case of tDCS), consistent with prior studies (Nitsche and Paulus, 2000; Terney et al., 2008; Bastani and Jaberzadeh, 2012; Jooss et al., 2019; Ehrhardt et al., 2021; Hoshi et al., 2021). This configuration has been shown to be particularly effective for modulating excitability in M1 (Moliadze et al., 2010; Nitsche and Paulus, 2000). However, it is important to acknowledge that the orbitofrontal cortex–located beneath the reference electrode–plays a significant role in emotional processing (Rolls and Grabenhorst, 2008; Rolls, 2019), and may therefore be affected by the stimulation. Using subjective assessments of mood and emotional state would have allowed for the monitoring of potential confounding effects resulting from unintended modulation of the orbitofrontal cortex. Fifth, differences in perceived scalp sensation may have influenced motor performance. As shown in previous studies (Fertonani et al., 2015) and supported by our findings, anodal tDCS was associated with greater discomfort compared to tRNS. While tDCS delivers a constant current that continuously activates cutaneous receptors, tRNS uses fluctuating currents that are less likely to stimulate sensory fibers linked to discomfort. This heightened sensory input in the tDCS condition–independent of its neuromodulatory effects–may have negatively influenced participants’ performance. Moreover, instructing participants to report side effects and rate their discomfort shortly after stimulation onset may have heightened their awareness of these sensations, potentially exacerbating their impact on task performance. Notably, Fertonani et al. (2015) found that the perceived sensations during sham and real tRNS conditions were indistinguishable, whereas the difference between anodal tDCS and sham was marginally significant (*p* = 0.056). To mitigate these sensory confounds, a within-between design could be implemented. This would involve comparing two stimulation groups–direct current and random noise–each with appropriate sham-controlled, within-subjects conditions, to better isolate neuromodulatory effects from sensory artifacts. Lastly, incorporating subjective assessments of participants’ expectations and beliefs across the different conditions would have provided greater clarity regarding the potential contribution of placebo effects to motor performance.

### 4.2 Conclusion

Neither atDCS nor hf-tRNS, applied for 10 min at an amplitude of 1.0 mA over the right M1, significantly improved kinematic measures in healthy young participants compared to sham stimulation, and no significant differences were found between the two active conditions. Delta values between pretest and posttest did not differ between stimulation conditions. However, significant improvements in MT and PV from pretest to posttest were observed exclusively following tRNS. Although no statistically significant advantage was established for any specific stimulation condition, the findings suggest that tRNS may be associated with modest improvements in motor performance and merit further investigation. In addition, within this experimental setup, sex does not appear to influence the effects of NIBS on motor performance. These insights may contribute to the application of tDCS and tRNS in neurorehabilitation settings.

## Data Availability

The raw data supporting the conclusions of this article will be made available by the authors, without undue reservation.

## References

[B1] Abe T. Miyaguchi S. Otsuru N. Onishi H. (2019). The effect of transcranial random noise stimulation on corticospinal excitability and motor performance. *Neurosci. Lett.* 705 138–142. 10.1016/j.neulet.2019.04.049 31028846

[B2] Adenzato M. Manenti R. Gobbi E. Enrici I. Rusich D. Cotelli M. (2019). Aging, sex and cognitive theory of mind: A transcranial direct current stimulation study. *Sci. Rep.* 9:18064. 10.1038/s41598-019-54469-4 31792263 PMC6889494

[B3] Amunts K. Schlaug G. Schleicher A. Steinmetz H. Dabringhaus A. Roland P. E. (1996). Asymmetry in the human motor cortex and handedness. *NeuroImage* 4(3 Pt 1), 216–222. 10.1006/nimg.1996.0073 9345512

[B4] Antal A. Herrmann C. S. (2016). Transcranial alternating current and random noise stimulation: Possible mechanisms. *Neural. Plast.* 2016:3616807. 10.1155/2016/3616807 27242932 PMC4868897

[B5] Arnao V. Riolo M. Carduccio F. Tuttolomondo A. D’Amelio M. Brighina F. (2019). Effects of transcranial random noise stimulation combined with Graded Repetitive Arm Supplementary Program (GRASP) on motor rehabilitation of the upper limb in sub-acute ischemic stroke patients: A randomized pilot study. *J Neural. Transm.* 126 1701–1706. 10.1007/s00702-019-02087-9 31576424

[B6] Bastani A. Jaberzadeh S. (2012). Does anodal transcranial direct current stimulation enhance excitability of the motor cortex and motor function in healthy individuals and subjects with stroke: A systematic review and meta-analysis. *Clin. Neurophysiol.* 123 644–657. 10.1016/j.clinph.2011.08.029 21978654

[B7] Batsikadze G. Moliadze V. Paulus W. Kuo M. F. Nitsche M. A. (2013). Partially non-linear stimulation intensity-dependent effects of direct current stimulation on motor cortex excitability in humans. *J. Physiol.* 591 1987–2000. 10.1113/jphysiol.2012.249730 23339180 PMC3624864

[B8] Bhattacharjee S. Kashyap R. Goodwill A. M. O’Brien B. A. Rapp B. Oishi K. (2022). Sex difference in tDCS current mediated by changes in cortical anatomy: A study across young, middle and older adults. *Brain Stimul.* 15 125–140. 10.1016/j.brs.2021.11.018 34826627 PMC9041842

[B9] Bieck S. M. Artemenko C. Moeller K. Klein E. (2018). Low to no effect: Application of tRNS during two-digit addition. *Front. Neurosci.* 12:176. 10.3389/fnins.2018.00176 29674948 PMC5895770

[B10] Boggio P. S. Castro L. O. Savagim E. A. Braite R. Cruz V. C. Rocha R. R. (2006). Enhancement of non-dominant hand motor function by anodal transcranial direct current stimulation. *Neurosci. Lett.* 404 232–236. 10.1016/j.neulet.2006.05.051 16808997

[B11] Braga M. Barbiani D. Emadi Andani M. Villa-Sánchez B. Tinazzi M. Fiorio M. (2021). The role of expectation and beliefs on the effects of non-invasive brain stimulation. *Brain Sci.* 11:1526. 10.3390/brainsci11111526 34827526 PMC8615662

[B12] Brevet-Aeby C. Mondino M. Poulet E. Brunelin J. (2019). Three repeated sessions of transcranial random noise stimulation (tRNS) leads to long-term effects on reaction time in the Go/No Go task. *Neurophysiol. Clin.* 49 27–32. 10.1016/j.neucli.2018.10.066 30414823

[B13] Broeder S. Nackaerts E. Heremans E. Vervoort G. Meesen R. Verheyden G. (2015). Transcranial direct current stimulation in Parkinson’s disease: Neurophysiological mechanisms and behavioral effects. *Neurosci. Biobehav. Rev.* 57 105–117. 10.1016/j.neubiorev.2015.08.010 26297812

[B14] Butler A. J. Shuster M. O’Hara E. Hurley K. Middlebrooks D. Guilkey K. (2013). A meta-analysis of the efficacy of anodal transcranial direct current stimulation for upper limb motor recovery in stroke survivors. *J. Hand Ther.* 26 162–171. 10.1016/j.jht.2012.07.002 22964028

[B15] Chaieb L. Antal A. Paulus W. (2015). Transcranial random noise stimulation-induced plasticity is NMDA-receptor independent but sodium-channel blocker and benzodiazepines sensitive. *Front. Neurosci.* 9:125. 10.3389/fnins.2015.00125 25914617 PMC4392589

[B16] Chaieb L. Paulus W. Antal A. (2011). Evaluating aftereffects of short-duration transcranial random noise stimulation on cortical excitability. *Neural Plast.* 2011:105927. 10.1155/2011/105927 21808744 PMC3144676

[B17] Charvet L. E. Dobbs B. Shaw M. T. Bikson M. Datta A. Krupp L. B. (2018). Remotely supervised transcranial direct current stimulation for the treatment of fatigue in multiple sclerosis: Results from a randomized, sham-controlled trial. *Mult. Scler.* 24 1760–1769. 10.1177/1352458517732842 28937310 PMC5975187

[B18] Chew T. Ho K. A. Loo C. K. (2015). Inter-and intra-individual variability in response to transcranial direct current stimulation (tDCS) at varying current intensities. *Brain Stimul.* 8 1130–1137. 10.1016/j.brs.2015.07.031 26294061

[B19] Dubljević V. Saigle V. Racine E. (2014). The rising tide of tDCS in the media and academic literature. *Neuron* 82 731–736. 10.1016/j.neuron.2014.05.003 24853934

[B20] Ehrhardt S. E. Filmer H. L. Wards Y. Mattingley J. B. Dux P. E. (2021). The influence of tDCS intensity on decision-making training and transfer outcomes. *J. Neurophysiol.* 125 385–397. 10.1152/jn.00423.2020 33174483

[B21] Elsner B. Kugler J. Pohl M. Mehrholz J. (2020). Transcranial direct current stimulation (tDCS) for improving activities of daily living, and physical and cognitive functioning, in people after stroke. *Cochrane Database Syst. Rev.* 11:CD009645. 10.1002/14651858.cd009645.pub4 33175411 PMC8095012

[B22] Esmaeilpour Z. Marangolo P. Hampstead B. M. Bestmann S. Galletta E. Knotkovam H. (2018). Incomplete evidence that increasing current intensity of tDCS boosts outcomes. *Brain Stimul.* 11 310–321. 10.1016/j.brs.2017.12.002 29258808 PMC7050474

[B23] Fehring D. J. Samandra R. Haque Z. Z. Jaberzadeh S. Rosa M. Mansouri F. A. (2021). Investigating the sex-dependent effects of prefrontal cortex stimulation on response execution and inhibition. *Biol. Sex Differ.* 12:47. 10.1186/s13293-021-00390-3 34404467 PMC8369781

[B24] Fertonani A. Ferrari C. Miniussi C. (2015). What do you feel if I apply transcranial electric stimulation? Safety, sensations and secondary induced effects. *Clin. Neurophysiol.* 126 2181–2188. 10.1016/j.clinph.2015.03.015 25922128

[B25] Gennatas E. D. Avants B. B. Wolf D. H. Satterthwaite T. D. Ruparel K. Ciric R. (2017). Age-Related effects and sex differences in gray matter density, volume, mass, and cortical thickness from childhood to young adulthood. *J. Neurosci.* 37 5065–5073. 10.1523/JNEUROSCI.3550-16.2017 28432144 PMC5444192

[B26] Ghilardi M. F. Moisello C. Silvestri G. Ghez C. Krakauer J. W. (2009). Learning of a sequential motor skill comprises explicit and implicit components that consolidate differently. *J. Neurophysiol.* 101 2218–2229. 10.1152/jn.01138.2007 19073794 PMC2681421

[B27] Ghilardi M. Ghez C. Dhawan V. Moeller J. Mentis M. Nakamura T. (2000). Patterns of regional brain activation associated with different forms of motor learning. *Brain Res.* 871 127–145. 10.1016/s0006-8993(00)02365-9 10882792

[B28] Gorbet D. J. Staines W. R. (2011). Inhibition of contralateral premotor cortex delays visually guided reaching movements in men but not in women. *Exp. Brain Res.* 212 315–325. 10.1007/s00221-011-2731-y 21607701

[B29] Haeckert J. Lasser C. Pross B. Hasan A. Strube W. (2020). Comparative study of motor cortical excitability changes following anodal tDCS or high-frequency tRNS in relation to stimulation duration. *Physiol. Rep.* 8:e14595. 10.14814/phy2.14595 32996722 PMC7525483

[B30] Hanlon C. A. McCalley D. M. (2022). Sex/gender as a factor that influences transcranial magnetic stimulation treatment outcome: Three potential biological explanations. *Front. Psychiatry* 13:869070. 10.3389/fpsyt.2022.869070 35573331 PMC9098922

[B31] Hayward K. S. Brauer S. G. Ruddy K. L. Lloyd D. Carson R. G. (2017). Repetitive reaching training combined with transcranial random noise stimulation in stroke survivors with chronic and severe arm paresis is feasible: A pilot, triple-blind, randomised case series. *J. Neuroeng. Rehabil.* 14:46. 10.1186/s12984-017-0253-y 28558789 PMC5450344

[B32] Herpich F. Contò F. van Koningsbruggen M. Battelli L. (2018). Modulating the excitability of the visual cortex using a stimulation priming paradigm. *Neuropsychologia* 119 165–171. 10.1016/j.neuropsychologia.2018.08.009 30107155

[B33] Herpich F. Melnick M. D. Agosta S. Huxlin K. R. Tadin D. Battelli L. (2019). Boosting learning efficacy with noninvasive brain stimul in intact and brain-damaged humans. *J. Neurosci.* 39 5551–5561. 10.1523/JNEUROSCI.3248-18.2019 31133558 PMC6616291

[B34] Ho K. A. Taylor J. L. Loo C. K. (2015). Comparison of the effects of transcranial random noise stimulation and transcranial direct current stimulation on motor cortical excitability. *J. ECT* 31 67–72. 10.1097/YCT.0000000000000155 25010032

[B35] Hoshi H. Kojima S. Otsuru N. Onishi H. (2021). Effects of transcranial random noise stimulation timing on corticospinal excitability and motor function. *Behav. Brain Res.* 414:113479. 10.1016/j.bbr.2021.113479 34302882

[B36] Hsu G. Jafari Z. H. Ahmed A. Edwards D. J. Cohen L. G. Parra L. C. (2025). Dose–response of tDCS effects on motor learning and cortical excitability: A preregistered study. *Imag. Neurosci.* 3:imag_a_00431. 10.1162/imag_a_00431 PMC12319989 40800917

[B37] Inghilleri M. Conte A. Currà A. Frasca V. Lorenzano C. Berardelli A. (2004). Ovarian hormones and cortical excitability. An rTMS study in humans. *Clin. Neurophysiol.* 115 1063–1068. 10.1016/j.clinph.2003.12.003 15066531

[B38] Inukai Y. Saito K. Sasaki R. Tsuiki S. Miyaguchi S. Kojima S. (2016). Comparison of three non-invasive transcranial electrical stimulation methods for increasing cortical excitability. *Front. Hum. Neurosci.* 10:668. 10.3389/fnhum.2016.00668 28082887 PMC5186778

[B39] Jobgen W. Meininger C. J. Jobgen S. C. Li P. Lee M. J. Smith S. B. (2009). Dietary l-arginine supplementation reduces white fat gain and enhances skeletal muscle and brown fat masses in diet-induced obese rats. *J. Nutr.* 139 230–237. 10.3945/jn.108.096362 19106310 PMC3151442

[B40] Jooss A. Haberbosch L. Köhn A. Rönnefarth M. Bathe-Peters R. Kozarzewski L. (2019). Motor task-dependent dissociated effects of transcranial random noise stimulation in a finger-tapping task versus a Go/No-Go task on corticospinal excitability and task performance. *Front. Neurosci.* 13:16131. 10.3389/fnins.2019.0016131 PMC6400855 30872997

[B41] Kang N. Summers J. J. Cauraugh J. H. (2016). Transcranial direct current stimulation facilitates motor learning post-stroke: A systematic review and meta-analysis. *J. Neurol. Neurosurg. Psychiatry* 87 345–355. 10.1136/jnnp-2015-311242 26319437

[B42] Kim S. Stephenson M. C. Morris P. G. Jackson S. R. (2014). tDCS-induced alterations in GABA concentration within primary motor cortex predict motor learning and motor memory: A 7 T magnetic resonance spectroscopy study. *Neuroimage* 99 237–243. 10.1016/j.neuroimage.2014.05.070 24904994 PMC4121086

[B43] Kortuem V. Kadish N. E. Siniatchkin M. Moliadze V. (2019). Efficacy of tRNS and 140 Hz tACS on motor cortex excitability seemingly dependent on sensitivity to sham stimulation. *Exp. Brain Res.* 237 2885–2895. 10.1007/s00221-019-05640-w 31482197

[B44] Krause B. Cohen Kadosh R. (2014). Not all brains are created equal: The relevance of individual differences in responsiveness to transcranial electrical stimulation. *Front. Syst. Neurosci.* 8:25. 10.3389/fnsys.2014.00025 24605090 PMC3932631

[B45] Kuo M. F. Paulus W. Nitsche M. A. (2006). Sex differences in cortical neuroplasticity in humans. *Neuroreport* 17 1703–1707. 10.1097/01.wnr.0000239955.68319.c2 17047457

[B46] Laakso I. Tanaka S. Koyama S. Saints V. D. Hirata A. (2015). Inter-subject variability in electric fields of motor cortical tDCS. *Brain Stimul.* 8 906–913. 10.1016/j.brs.2015.05.002 26026283

[B47] Laczó B. Antal A. Rothkegel H. Paulus W. (2014). Increasing human leg motor cortex excitability by transcranial high frequency random noise stimulation. *Restor. Neurol. Neurosci.* 32 403–410. 10.3233/RNN-130367 24576783

[B48] Lerner O. Friedman J. Frenkel-Toledo S. (2021). The effect of high-definition transcranial direct current stimulation intensity on motor performance in healthy adults: A randomized controlled trial. *J. Neuroeng. Rehabil.* 18:103. 10.1186/s12984-021-00899-z 34174914 PMC8236155

[B49] Lopez-Alonso V. Cheeran B. Rio-Rodriguez D. Fernandez-del-Olmo M. (2014). Inter-individual variability in response to non-invasive brain stimulation paradigms. *Brain Stimul.* 7 372–380. 10.1016/j.brs.2014.02.004 24630849

[B50] Lopez-Alonso V. Fernandez-del-Olmo M. Costantini A. Gonzalez-Henriquez J. J. Cheeran B. (2015). Intra-individual variability in the response to anodal transcranial direct current stimulation. *Clin. Neurophysiol.* 126 2342–2347. 10.1016/j.clinph.2015.03.022 25922127

[B51] Luders E. Narr K. L. Thompson P. M. Rex D. E. Jancke L. Steinmetz H. (2004). Gender differences in cortical complexity. *Nat. Neurosci.* 7 799–800. 10.1038/nn1277 15338563

[B52] Ma W. Wang F. Yi Y. Huang Y. Li X. Liu Y. (2024). Mapping the electric field of high-definition transcranial electrical stimulation across the lifespan. *Sci. Bull.* 69 3876–3888. 10.1016/j.scib.2024.10.001 39424454

[B53] Manippa V. Padulo C. van der Laan L. N. Brancucci A. (2017). Gender differences in food choice: Effects of superior temporal sulcus stimulation. *Front. Hum. Neurosci.* 11:597. 10.3389/fnhum.2017.00597 29270120 PMC5725471

[B54] Masina F. Arcara G. Galletti E. Cinque I. Gamberini L. Mapelli D. (2021). Neurophysiological and behavioural effects of conventional and high definition tDCS. *Sci. Rep.* 11:7659. 10.1038/s41598-021-87371-z 33828202 PMC8027218

[B55] McDonnell M. D. Abbott D. (2009). What is stochastic resonance? Definitions, misconceptions, debates, and its relevance to biology. *PLoS Comput. Biol.* 5:e1000348. 10.1371/journal.pcbi.1000348 19562010 PMC2660436

[B56] Miniussi C. Harris J. A. Ruzzoli M. (2013). Modelling non-invasive brain stimulation in cognitive neuroscience. *Neurosci. Biobehav. Rev.* 37 1702–1712. 10.1016/j.neubiorev.2013.06.014 23827785

[B57] Moisello C. Crupi D. Tunik E. Quartarone A. Bove M. Tononi G. (2009). The serial reaction time task revisited: A study on motor sequence learning with an arm-reaching task. *Exp. Brain Res.* 194 143–155. 10.1007/s00221-008-1681-5 19104787 PMC2804101

[B58] Moliadze V. Antal A. Paulus W. (2010). Electrode-distance dependent after-effects of transcranial direct and random noise stimulation with extracephalic reference electrodes. *Clin. Neurophysiol*. 121, 2165–2171. 10.1016/j.clinph.2010.04.033 20554472

[B59] Moliadze V. Atalay D. Antal A. Paulus W. (2012). Close to threshold transcranial electrical stimulation preferentially activates inhibitory networks before switching to excitation with higher intensities. *Brain Stimul.* 5 505–511. 10.1016/j.brs.2011.11.004 22445135

[B60] Moliadze V. Fritzsche G. Antal A. (2014). Comparing the efficacy of excitatory transcranial stimulation methods measuring motor evoked potentials. *Neural. Plast.* 2014:837141. 10.1155/2014/837141 24804104 PMC3997131

[B61] Moliadze V. Schmanke T. Andreas S. Lyzhko E. Freitag C. M. Siniatchkin M. (2015). Stimulation intensities of transcranial direct current stimulation have to be adjusted in children and adolescents. *Clin. Neurophysiol.* 126 1392–1399. 10.1016/j.clinph.2014.10.14224 25468234

[B62] Monastero R. Baschi R. Nicoletti A. Pilati L. Pagano L. Cicero C. E. (2020). Transcranial random noise stimulation over the primary motor cortex in PD-MCI patients: A crossover, randomized, sham-controlled study. *J. Neural Transm.* 127 1589–1597. 10.1007/s00702-020-02255-2 32965593 PMC7666273

[B63] Monte-Silva K. Kuo M. F. Liebetanz D. Paulus W. Nitsche M. A. (2010). Shaping the optimal repetition interval for cathodal transcranial direct current stimulation (tDCS). *J. Neurophysiol.* 103 1735–1740. 10.1152/jn.00924.2009 20107115

[B64] Moret B. Donato R. Nucci M. Cona G. Campana G. (2019). Transcranial random noise stimulation (tRNS): A wide range of frequencies is needed for increasing cortical excitability. *Sci. Rep.* 9:15150. 10.1038/s41598-019-51553-7 31641235 PMC6806007

[B65] Moss F. Ward L. M. Sannita W. G. (2004). Stochastic resonance and sensory information processing: A tutorial and review of application. *Clin. Neurophysiol.* 115 267–281. 10.1016/j.clinph.2003.09.014 14744566

[B66] Nitsche M. A. Paulus W. (2000). Excitability changes induced in the human motor cortex by weak transcranial direct current stimulation. *J. Physiol.* 527(Pt 3), 633–639. 10.1111/j.1469-7793.2000.t01-1-00633.x 10990547 PMC2270099

[B67] Nuzum N. D. Hendy A. M. Russell A. P. Teo W. P. (2016). Measures to predict the individual variability of corticospinal responses following transcranial direct current stimulation. *Front. Hum. Neurosci.* 10:487. 10.3389/fnhum.2016.00487 27766075 PMC5052268

[B68] O’Brien A. T. Bertolucci F. Torrealba-Acosta G. Huerta R. Fregni F. Thibaut A. (2018). Non-invasive Brain Stimul for fine motor improvement after stroke: A meta-analysis. *Eur. J. Neurol.* 25 1017–1026. 10.1111/ene.13643 29744999

[B69] Oldfield R. C. (1971). The assessment and analysis of handedness: The Edinburgh inventory. *Neuropsychologia* 9 97–113. 10.1016/0028-3932(71)90067-4 5146491

[B70] Pascual-Leone A. Cohen L. G. Brasil-Neto J. P. Hallett M. (1994). Non-invasive differentiation of motor cortical representation of hand muscles by mapping of optimal current directions. *Electroencephalogr. Clin. Neurophysiol.* 93 42–48. 10.1016/0168-5597(94)90090-6 7511521

[B71] Patel R. Ashcroft J. Patel A. Ashrafian H. Woods A. J. Singh H. (2019). The impact of transcranial direct current stimulation on upper-limb motor performance in healthy adults: A systematic review and meta-analysis. *Front. Neurosci.* 13:1213. 10.3389/fnins.2019.01213 31803003 PMC6873898

[B72] Pavan A. Ghin F. Contillo A. Milesi C. Campana G. Mather G. (2019). Modulatory mechanisms underlying high-frequency transcranial random noise stimulation (hf-tRNS): A combined stochastic resonance and equivalent noise approach. *Brain Stimul.* 12 967–977. 10.1016/j.brs.2019.02.018 30833217

[B73] Peterchev A. V. Wagner T. A. Miranda P. C. Nitsche M. A. Paulus W. Lisanby S. H. (2012). Fundamentals of transcranial electric and magnetic stimulation dose: Definition, selection, and reporting practices. *Brain Stimul.* 5 435–453. 10.1016/j.brs.2011.10.001 22305345 PMC3346863

[B74] Peters H. T. Edwards D. J. Wortman-Jutt S. Page S. J. (2016). Moving forward by stimulating the brain: Transcranial direct current stimulation in post-stroke hemiparesis. *Front. Hum. Neurosci.* 10:394. 10.3389/fnhum.2016.00394 27555811 PMC4977294

[B75] Prichard G. Weiller C. Fritsch B. Reis J. (2014). Effects of different electrical brain stimul protocols on subcomponents of motor skill learning. *Brain Stimul.* 7 532–540. 10.1016/j.brs.2014.04.005 24810956

[B76] Rampersad S. Stegeman D. Oostendorp T. (2011). On handling the layered structure of the skull in transcranial direct current stimulation models. *Annu. Int. Conf. IEEE Eng. Med. Biol. Soc.* 2011 1989–1992. 10.1109/IEMBS.2011.6090560 22254724

[B77] Reis J. Fritsch B. (2011). Modulation of motor performance and motor learning by transcranial direct current stimulation. *Curr. Opin. Neurol.* 24 590–596. 10.1097/WCO.0b013e32834c3db0 21968548

[B78] Rolls E. T. (2019). The orbitofrontal cortex and emotion in health and disease, including depression. *Neuropsychologia* 128 14–43. 10.1016/j.neuropsychologia.2017.09.021 28951164

[B79] Rolls E. T. Grabenhorst F. (2008). The orbitofrontal cortex and beyond: From affect to decision-making. *Prog. Neurobiol.* 86 216–244. 10.1016/j.pneurobio.2008.09.001 18824074

[B80] Rudroff T. Workman C. D. Fietsam A. C. Kamholz J. (2020). Response variability in transcranial direct current stimulation: Why sex matters. *Front. Psychiatry* 11:585. 10.3389/fpsyt.2020.00585 32636774 PMC7316984

[B81] Ruigrok A. N. Salimi-Khorshidi G. Lai M. C. Baron-Cohen S. Lombardo M. V. Tait R. J. (2014). A meta-analysis of sex differences in human brain structure. *Neurosci. Biobehav. Rev.* 39 34–50. 10.1016/j.neubiorev.2013.12.004 24374381 PMC3969295

[B82] Russell M. J. Goodman T. A. Visse J. M. Beckett L. Saito N. Lyeth B. G. (2017). Sex and electrode configuration in transcranial electrical stimulation. *Front. Psychiatry* 8:147. 10.3389/fpsyt.2017.00147 28855877 PMC5558260

[B83] Russell M. Goodman T. Wang Q. Groshong B. Lyeth B. G. (2014). Gender differences in current received during transcranial electrical stimulation. *Front. Psychiatry* 5:104. 10.3389/fpsyt.2014.00104 25177301 PMC4133690

[B84] Saiote C. Polanía R. Rosenberger K. Paulus W. Antal A. (2013). High-frequency tRNS reduces BOLD activity during visuomotor learning. *PLoS One* 8:e59669. 10.1371/journal.pone.0059669 23527247 PMC3603861

[B85] Sánchez-Kuhn A. Pérez-Fernández C. Cánovas R. Flores P. Sánchez-Santed F. (2017). Transcranial direct current stimulation as a motor neurorehabilitation tool: An empirical review. *Biomed. Eng. Online* 16 (Suppl. 1):76. 10.1186/s12938-017-0361-8 28830433 PMC5568608

[B86] Sánchez-León C. A. Sánchez-López A. Gómez-Climent M. A. Cordones I. Kadosh R. C. Márquez-Ruiz J. (2021). Impact of chronic transcranial random noise stimulation (tRNS) on GABAergic and glutamatergic activity markers in the prefrontal cortex of juvenile mice. *Prog. Brain Res.* 264 323–341. 10.1016/bs.pbr.2021.01.017 34167661

[B87] Sheffield J. G. Ramerpresad S. Brem A. K. Mansfield K. Orhan U. Dillard M. (2022). Blinding efficacy and adverse events following repeated transcranial alternating current, direct current, and random noise stimulation. *Cortex* 154 77–88. 10.1016/j.cortex.2022.05.015 35759817

[B88] Smith M. J. Adams L. F. Schmidt P. J. Rubinow D. R. Wassermann E. M. (2002). Effects of ovarian hormones on human cortical excitability. *Ann. Neurol.* 51 599–603. 10.1002/ana.10180 12112106

[B89] Splittgerber M. Suwelack J. H. Kadish N. E. Moliadze V. (2020). The effects of 1 mA tACS and tRNS on children/adolescents and adults: Investigating age and sensitivity to sham stimulation. *Neural Plast.* 2020:8896423. 10.1155/2020/8896423 32855633 PMC7443018

[B90] Stagg C. J. Antal A. Nitsche M. A. (2018). Physiology of transcranial direct current stimulation. *J. ECT* 34 144–152. 10.1097/YCT.0000000000000510 29877965

[B91] Strube W. Bunse T. Malchow B. Hasan A. (2015). Efficacy and interindividual variability in motor-cortex plasticity following anodal tDCS and pairedassociative stimulation. *Neural Plast.* 2015:530423. 10.1155/2015/530423 25866683 PMC4381571

[B92] Strube W. Bunse T. Nitsche M. A. Nikolaeva A. Palm U. Padberg F. (2016). Bidirectional variability in motor cortex excitability modulation following 1 mA transcranial direct current stimulation in healthy participants. *Physiol. Rep.* 4:e12884. 10.14814/phy2.12884 27495298 PMC4985549

[B93] Swissa Y. Hacohen S. Friedman J. Frenkel-Toledo S. (2022). Sensorimotor performance after high-definition transcranial direct current stimulation over the primary somatosensory or motor cortices in men versus women. *Sci. Rep.* 12:11117. 10.1038/s41598-022-15226-2 35778465 PMC9249866

[B94] Tedesco Triccas L. Burridge J. H. Hughes A. M. Pickering R. M. Desikan M. Rothwell J. C. (2016). Multiple sessions of transcranial direct current stimulation and upper extremity rehabilitation in stroke: A review and meta-analysis. *Clin. Neurophysiol.* 127 946–955. 10.1016/j.clinph.2015.04.067 25998205

[B95] Terney D. Chaieb L. Moliadze V. Antal A. Paulus W. (2008). Increasing human brain excitability by transcranial high-frequency random noise stimulation. *J. Neurosci.* 28 14147–14155. 10.1523/JNEUROSCI.4248-08.2008 19109497 PMC6671476

[B96] Thomas C. Ghodratitoostani I. Delbem A. C. B. Ali A. Datta A. (2019). Influence of gender-related differences in transcranial direct current stimulation: A computational study. *Annu. Int. Conf. IEEE Eng. Med. Biol. Soc.* 2019 5196–5199. 10.1109/EMBC.2019.8856898 31947029

[B97] Van Hoornweder S. Vanderzande L. Bloemers E. Verstraelen S. Depestele S. Cuypers K. (2021). The effects of transcranial direct current stimulation on upper-limb function post-stroke: A meta-analysis of multiple-session studies. *Clin. Neurophysiol.* 132 1897–1918. 10.1016/j.clinph.2021.05.015 34157634

[B98] Vergallito A. Feroldi S. Pisoni A. Romero Lauro L. J. (2022). Inter-Individual variability in tDCS effects: A narrative review on the contribution of stable, variable, and contextual factors. *Brain Sci.* 12:522. 10.3390/brainsci12050522 35624908 PMC9139102

[B99] Ward L. M. (2009). Physics of neural synchronisation mediated by stochastic resonance. *Contemp. Phys.* 50 563–574. 10.1080/00107510902879246

[B100] Wei J. Carroll R. J. Harden K. K. Wu G. (2012). Comparisons of treatment means when factors do not interact in two-factorial studies. *Amino Acids* 42 2031–2035. 10.1007/s00726-011-0924-0 21547361 PMC3199378

[B101] Wiethoff S. Hamada M. Rothwell J. C. (2014). Variability in response to transcranial direct current stimulation of the motor cortex. *Brain Stimul.* 7 468–475. 10.1016/j.brs.2014.02.003 24630848

[B102] Yang T. Banissy M. J. (2017). Enhancing anger perception in older adults by stimulating inferior frontal cortex with high frequency transcranial random noise stimulation. *Neuropsychologia* 102 163–169. 10.1016/j.neuropsychologia.2017.06.017 28625658

[B103] Yao J. Li X. Zhang W. Lin X. Lyu X. Lou W. (2021). Analgesia induced by anodal tDCS and high-frequency tRNS over the motor cortex: Immediate and sustained effects on pain perception. *Brain Stimul.* 14 1174–1183. 10.1016/j.brs.2021.07.011 34371209

[B104] Zhang M. Cheng I. Sasegbon A. Dou Z. Hamdy S. (2021). Exploring parameters of gamma transcranial alternating current stimulation (tACS) and full-spectrum transcranial random noise stimulation (tRNS) on human pharyngeal cortical excitability. *Neurogastroenterol. Motil.* 33:e14173. 10.1111/nmo.14173 34081376

